# Primary pulmonary amebic abscess in a patient with pulmonary adenocarcinoma: a case report

**DOI:** 10.1186/s40249-018-0419-2

**Published:** 2018-04-27

**Authors:** Yuan-Yuan Liu, Yue Ying, Chong Chen, Yue-Kai Hu, Fei-Fei Yang, Ling-Yun Shao, Xun-Jia Cheng, Yu-Xian Huang

**Affiliations:** 1 Department of Infectious Diseases, Huashan Hospital, Fudan University, 12, Middle Wulumuqi Road, Shanghai, 200040 China; 2 Department of Infectious Diseases, Chongqing Kaixian People’s Hospital, 8, Ankang Road, Chongqing, 405499 China; 3 Department of Medical Microbiology and Parasitology, School of Basic Medical Sciences, Fudan University, 130 Dongan Road, Shanghai, 200032 China

**Keywords:** Amebiasis, Pulmonary amebic abscess, Pulmonary adenocarcinoma

## Abstract

**Background:**

Primary pulmonary amoeba is very rare and here we report a case of a 68-year-old man presenting with primary pulmonary amoeba after undergoing chemotherapy for lung adenocarcinoma.

**Case presentation:**

In October 2016, the man aged 68 was admitted to our hospital because of repeated cough for 8 months and hemoptysis for 1 month. He was diagnosed lung adenocarcinoma and underwent surgery in 2012 without receiving chemotherapy. In March 2016, the patients suffered recurrence of cancer and was treated with chemotherapy. After 2 months of chemotherapy, the patient had consistent cough with white sputum, and chest CT showed a local lung nodule. The physicians suspected that the patient had pulmonary infectious diseases, and he was treated with empirical antibacterial treatment. However, his symptom wasn’t relieved and later the percutaneous lung biopsy found trophozites of *Entamoeba histolytica*. After administration of metronidazole, the symptoms of the patient were markedly relieved and the lesions were absorbed.

**Conclusions:**

In such cases where patients with pulmonary nodules were in immunodeficiency state and had adequate but ineffective anti-bacterial treatment, *Entamoeba histolytica* infection could be one of the rare causes. Percutaneous lung biopsy should be recommended and specific dying for parasites should be done when necessary.

**Electronic supplementary material:**

The online version of this article (10.1186/s40249-018-0419-2) contains supplementary material, which is available to authorized users.

## Multilingual abstracts

Please see Additional file 1 for translations of the abstract into the five official working languages of the United Nations.

## Background

Amebiasis is caused by *Entamoeba histolytica* (*E. histolytica*). Amebic liver abscess represents the most common manifestation of extraintestinal amebiasis, while pulmonary primary amoeba protozoa infection is usually rare and often occurs concurrently with amebic colitis or amebic liver abscess [1, 2]. Here we report a rare case of primary pulmonary amebic abscess without amebic colitis and amebic liver abscess.

## Case presentation

In October 2016, the man aged 68 was admitted to our hospital because of repeated cough for 8 months and hemoptysis for 1 month.

**Fig. 1:**
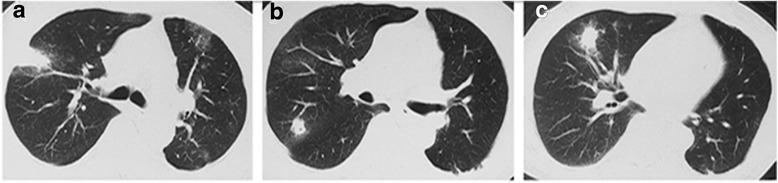
Pulmonary CT scan revealing multiple nodules in bilateral lung, including right upper lobe (**a** and **b**) and right middle lobe (**c**), which proved recurrence of lung cancer later by biopsy. (2016-3-21)

**Fig. 2:**
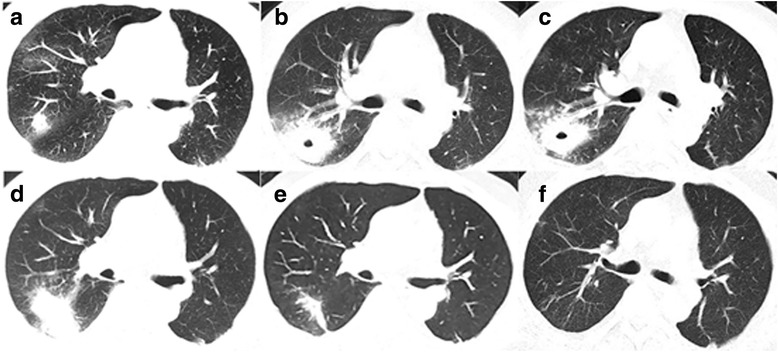
The patient’s pulmonary CT scan showed changes of the lesion during course of disease. **a** Right upper lobe nodule revealing recurrence of lung cancer confirmed by pathology (2016-3-21); **b** Right lung lesion became larger accompanied by an empty cavity after chemotherapy (2016-5-4); **c** No significant change of the right lung lesion after levofloxacin and cefepime treatment (2016-5-18); **d** No significant change of the right lung lesion after praziquantel treatment (2016-9-5); **e** Absorbed lesions after metronidazole treatment (2016-12-26); **f** No recurrence of amoeba during 6-month follow-up (2017-06-06)

The patient was diagnosed left lower lung adenocarcinoma (pT1N0M0, Ia) in 2012, and then received surgical resection without chemotherapy. In March 2016, the patient began to develop dry cough, without fever, chest pain, hemoptysis and night sweats. Chest computed tomography (CT) scan was performed and revealed multiple lung nodules, enlarged right hilar and mediastinal lymph nodes (Figs. 1 and 2a). Suspecting metastatic malignant tumor, further bronchoscopy biopsy was then performed, and the pathological diagnosis reported non-mucoid adenocarcinoma. At that time, the patient was diagnosed left lung adenocarcinoma recurrence (rT4N2M1, Ia), and underwent “pemetrexed + cisplatin” chemotherapy on March 23 and April 13. However, the patient still experienced cough, with white sputum and no hemoptysis. On May 4, the second chest CT showed that the right upper lobe nodules became larger in size, accompanied by an empty cavity while the remaining multiple nodules in both lungs have been absorbed (Fig. 2b). At that time, the right upper lobe lesion was considered infectious lesion and repeated sputum smear and culture were taken, all coming back negative, including acid-fast staining. The patient was then empirically treated with levofloxacin and cefepime for 7 d, but his symptoms didn’t relieve. On May 18, there was no change of the upper lobe lesion in a reviewed chest CT (Fig. 2c). The bronchoscopy biopsy was performed again and neither malignant tumor cells nor suspected pathogens were found. Moxifloxacin was administered for 7 d with no symptoms relief.

On July 28 2016, the patient underwent percutaneous right upper lung mass biopsy and pathological result found pulmonary consolidation with fibrosis containing parasitic eggs, suspecting paragonimiasis. Serum parasite antibodies including paragonimiasis, echinococcosis and schistosome antibodies were all negative and no eggs were found in stool. Praziquantel was given for 5 d with no improvement in symptoms.

On September 5 2016, the patient had hemoptysis and was transferred to our hospital. The patient denied a history of having raw or unclean diet and had a history of smoking for more than 30 years, occasionally drinking alcohol. He was born in rural area of Shanghai and had no travel history before the onset. He is heterosexual, married and has given birth. He has no malnutrition. Lung auscultation didn’t detect obvious wet rales. Laboratory examinations revealed normal blood routine (white blood cell: 5.59 × 10^9^/L, Neutrocyte %: 71.7%, Eosinophil %: 1.8%), slightly elevated erythrocyte sedimentation rate (ESR, 40 mm/h), normal tumor markers like carcinoembryonic antigen (CEA), neuron-specific enolase (NSE), cytokeratin 211 (CY211), and normal stool routine without parasitic eggs.

**Fig. 3:**
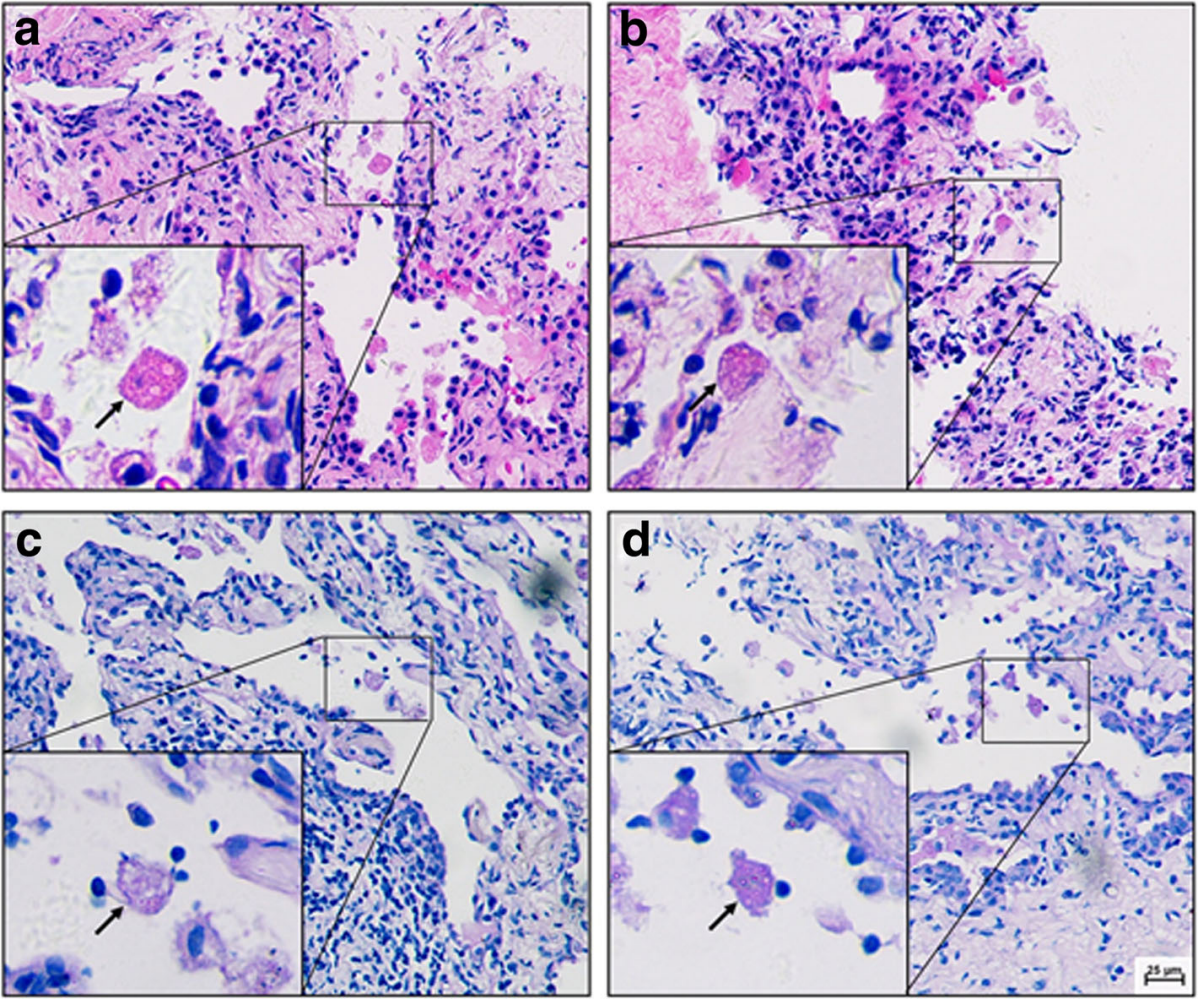
Pathological examination of the lung revealed trophozoites of *Entamoeba histolytica*. **a**, **b** Haematoxylin Eosin (HE) stain; **c**, **d** Periodic acid–Schiff (PAS) stain; arrows refer to the amoebic trophozoites)

**Fig. 4:**
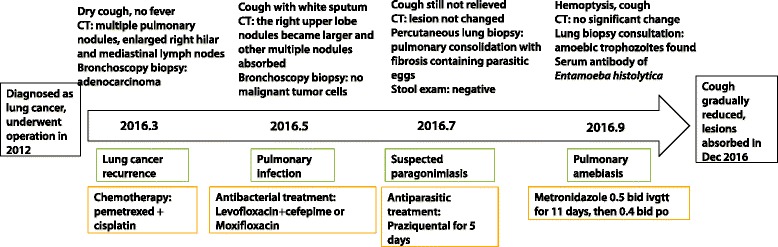
The timeline of the patient’s medical history, diagnostic workup and treatments

There was still no significant change of the right lung lesion in a reviewed chest CT examination (Fig. 2d). Still suspecting a possible parasite infection, the lung biopsy specimens were sent to Shanghai Medical College Institute of Parasitology for consultant. Amoebic trophozoites ingesting erythrocytes were found under the microscope (Fig. 3), and the serum antibody of amoebic trophozoites [3] was 1:64 (Additional file 2: Figure S1). The enteroscope examination showed multiple polyps. The PCR test for *Entamoeba gingivalis* was negative. The patient was diagnosed as pulmonary amebiasis and he was given metronidazole 0.5 bid ivgtt for 11 days, and then metronidazole 0.4 tid po before discharge. The patient’s cough gradually reduced and lesions were significantly absorbed by reviewed chest CT on December 26 (Fig. 2e). On June 6 2017, reviewed lung CT showed that the lesions were completely absorbed (Figs. 2f and 4).

## Discussion

Amebiasis is caused by *Entamoeba histolytica*. Up to 90% of patients infected with ameba may be asymptomatic [4]. The two most common manifestations of amebiasis are amebic colitis and amebic liver abscess [5, 6]. The prevalence of amebiasis was highest in areas that have poor sanitation, like Southeast Asia. In China, the disease rate was relevantly lower. However, China is a developing country where sanitation still fails in many districts, so the prevalence rate of amoebiasis in China was still higher than most developed countries. According to a research investigating 1312 serum samples throughout seven provinces in China, the positivity rate was 6.25% [3]. While in HIV-infected patients, the positivity rate reached 7.9% [7].

*Entamoeba histolytica* exist in two forms, trophozoites and cysts. The infection course is initiated by parasite adherence to mucus layer. Without effective defense from the host immune system, the colonized parasites can destruct host’s extracellular matrix by the pore-forming proteins and hydrolytic enzymes. Then the parasites penetrate into the portal circulation, reach liver and create the unique abscess. For the normal host immune response against amoebae, abundant IgA can prevent pathogens from adhering and penetrating the mucus layer. Intestinal epithelial cells can recognize the pathogens via toll-like receptor, then activate NF-κB to further produce inflammatory cytokines. Among these cytokines, Interferon-γ (IFN-γ) is the most important one involved in the clearance of infection [8]. Neutrophils and macrophages activated by IFN-γ can be attracted to the site of infection and produce reactive oxygen species (ROS) and nitric oxide (NO), playing critical roles in killing trophozoites [9]. Anti-neoplastic chemotherapy is often associated with profound immunosuppression and therefore an increased risk of infection. As neutrophils provide protection against a wide variety of opportunistic pathogens, the frequency of infections caused by these organisms is increased in patients with neutropenia. In this case, the patient underwent chemotherapy after suffering from a recurrence of lung adenocarcinoma. During chemotherapy, his neutrophil count reduced from 7.0 × 10^9^/L to 2.8 × 10^9^/L. While chest CT showed that most of the lesions of tumor recurrence disappeared, which indicated effective chemotherapy, one lesion grew larger. After adequate anti-bacterial therapy, his symptoms were not relieved and therefore, infection of the uncommon pathogens should be considered. Finally, amoeba trophozoites were found in the lung tissue biopsy specimens after multiple pathological reading, and the symptoms were improved significantly and the lung lesions were completely absorbed after metronidazole treatment.

Notably, this patient was characterized by solitary pulmonary lesion, without symptoms of intestinal infection and liver abscess. It was presumed to be a primary pulmonary amebic infection. According to a study conducted in the sever immunodeficient mice, neutrophils can play a protective role by physically containing the abscess [10]. We conjure that although the patient did not meet the diagnostic criteria of neutropenia, his neutrophils might have decreased function against infection in this case. And without enough functional neutrophils, the trophozoites may easily perforate the liver and directly reach the lung, forming primary lung abscess. However, the exact mechanism is still not clear and require further investigation.

In 2014, Zhu et al. [11] reported a case of pulmonary amoeba abscess with lung adenocarcinoma, which was diagnosed by finding amoeba trophozoites in the pleural effusion. The patient’s symptoms also recovered after treatment with anti-amebic agents, however, the patient in that study had a previous history of amebic infection, which was different from the case we reported.

## Conclusions

We concluded that in such cases where patients with pulmonary nodules were in immunodeficiency state and had adequate but ineffective anti-bacterial treatment, *Entamoeba histolytica* infection could be one of the rare causes. Percutaneous lung biopsy should be recommended and specific dying for parasites should be done when necessary to avoid misdiagnosis.

## Additional files

Additional file 1: Multilingual abstracts in the five official working languages of the United Nations. (PDF 561 kb)

Additional file 2: **Figure S1.** The serum antibody of amoebic trophozoites was positive by Immunofluorescence assay. A, Negative control; B, Serum of patient. (TIFF 2286 kb)

## Abbreviations

bid: Bis in die (Latin: twice a day)
CEA: Carcinoembryonic antigen
CT: Computed tomography
CY211: Cytokeratin 211
ESR: Erythrocyte sedimentation rate
IFN-γ: Interferon-γ
ivgtt: Intravenously guttae
NO: Nitric oxide
NSE: Neuron-specific enolase
po: Per os (Latin: by mouth, orally)
ROS: Reactive oxygen species
tid: Ter in die (Latin: three times a day)
