# Supplementary material for: Primary pulmonary amebic abscess in a patient with pulmonary adenocarcinoma: a case report
Source: Infect Dis Poverty. 2018 Apr 27;7:34. doi: 10.1186/s40249-018-0419-2 (PMC5921995; doi:10.1186/s40249-018-0419-2)
Supplement: Supplementary file 1 — Multilingual abstracts in the five official working languages of the United Nations. (PDF 561 kb) [file 40249_2018_419_MOESM1_ESM.pdf]

Translation of the abstract into the six official working languages of the United Nations

ظهور الخُراج الأميبي الرئوي الأولي لدى مريض يعاني من سرطان الغدة الرئوي:تقرير عن الحالة

قدمه يوان يوان ليو، وتشونغ تشن، ويوي-كاي هو، وفاي فاي يانغ، ولينغ يون شاو، وشون جيا تشنغ، ويو شيان هوانغ

الخلاصة

المعلومات الأساسية:إنَّ مرض الأميبا الرئوي الأولي نادرٌ جدًا وهنا نقدمُ تقريرًا عن حالة رجل يبلغ ٦٨ عاماً من العمر ويُظهر إصابته بمرض الأميبا الرئوي الأولي بعد خضوعه للعلاج الكيميائي لسرطان الغدة الرئوي.

عرض الحالة:ففي شهر أكتوبر ٢٠١٦م، أُدخل رجلٌ يبلغ ٦٨ عاماً من العمر إلى المستشفى الذي نعمل به بسبب حدوث حالة السُعال المتكرر لمدة ٨ أشهر مصحوبة ببصق للدم لمدة شهر. وتم تشخيص إصابته بسرطان الغدة الرئوي وخضع للجراحة في عام ٢٠١٢م دون تلقي العلاج الكيميائي. وفي شهر مارس ٢٠١٦م، عانى المريض من عودة السرطان وخضع للعلاج الكيميائي. وبعد شهرين من تلقي العلاج الكيميائي، عانى المريض من السُعال المستمر المصحوب ببلغم أبيض اللون، وأظهرت الأشعة المقطعية للصدر وجود عُقيدات موضعية بالرئة. اشتبه الأطباء بإصابة المريض بأمراض معدية بالدرنة، وتم علاجه باستخدام علاج تجريبي مضاد للبكتيريا. إلا أنّ ّه لم يُشفى من هذه الأعراض ولاحقاً أثبت الفحص بإبرة الخزعة من الرئة وجود نواشطتندرج لنوعالطفيليات المتحولة لحالة النُسُج. فبعد استخدام مركب المترونيدازول، شُفيت أعراض المريض بشكل ملحوظ واختفت الآفات.

النتائج:ففي حالات كهذه عندما يعاني المرضى من عُقيدات بالرئة بحالة قصور مناعي وخضعوا لعلاج كافٍ باستخدام مضاد للبكتيريا ولكنه لم يكن فعآلاً،فإن العدوى بالطفيليات المتحولة لحالة النُسُجقد تكون من إحدى المسببات النادرة الحدوث. ينبغي التوصية باجراء الفحص بإبرة الخزعة من الرئة ويجب القضاء على أنواع محددة من الطفيليات عند الحاجة لذلك.

Translated from English version into Arabic by Bashaier Allam, proofread by Heba Kandel, through

TRANSLATORS
WITHOUT BORDERS

肺腺癌患者原发性肺阿米巴脓肿 1 例

刘袁媛，应悦，陈冲，胡越凯，杨飞飞，邵凌云，程训佳，黄玉仙

摘要

引言：原发性肺阿米巴病非常少见，在此我们报道了一例 68 岁男性肺腺癌患者化疗后出现原发性肺阿米巴病。

病例介绍：2016 年 10 月，一位 68 岁男性患者因"反复咳嗽 8 月，咯血 1 月"入住我院。2012 年该患者被诊断为肺腺癌并接受手术治疗，术后未化疗。2016 年 3 月该患者肺腺癌复发，开始化疗。化疗 2 个月后患者出现持续咳嗽咳痰，胸部 CT 提示局灶肺结节。医生考虑为肺部感染，遂给予经验性抗感染治疗，然而患者症状并未减轻。随后患者接受经皮肺穿刺活检，在病灶部位发现溶组织阿米巴滋养体。经甲硝唑治疗后患者症状明显减轻，肺部病灶吸收。

结论：免疫缺陷患者若出现肺部新发结节，且经充分经验性抗感染治疗无效时，应考虑到少见的溶组织阿米巴感染。这类患者推荐使用经皮肺穿刺活检来诊断，必要时需做寄生虫相关的特殊检查。

Translated from English version into Chinese by Yuan-Yuan Liu

Abcès amibien pulmonaire primitif chez un patient porteur d'un adénocarcinome pulmonaire: rapport de cas

Yuan-Yuan Liu, Yue Ying, Chong Chen, Yue-Kai Hu, Fei-Fei Yang, Ling-Yun Shao, Xun-Jia Cheng, Yu-Xian Huang

Résumé

Contexte: L'amibiase pulmonaire primitive est une maladie très rare. Nous rapportons ici le cas d'un homme âgé de 68 ans présentant une amibiase pulmonaire primitive à la suite d'une chimiothérapie pour un adénocarcinome du poumon.

Présentation du cas: En octobre 2016, un homme âgé de 68 ans a été admis dans notre hôpital pour une toux récurrente depuis 8 mois et une hémoptysie depuis 1 mois. Nous lui avons diagnostiqué un adénocarcinome pulmonaire qui a été traité chirurgicalement en 2012, sans chimiothérapie. En mars 2016, ce patient a présenté une récidive du cancer et a été traité par chimiothérapie. Après deux mois de chimiothérapie, il s'est mis à tousser constamment, avec des expectorations blanches. Le scanner thoracique a révélé un nodule pulmonaire local. Les médecins ont suspecté une pneumopathie infectieuse et mis en place un traitement antibactérien empirique, qui n'a cependant pas fait régresser les symptômes. Par la suite, une biopsie percutanée du poumon a révélé des trophozoïtes d'*Entamoeba histolytica*. L'administration de métronidazole a permis de nettement atténuer les symptômes du patient et d'obtenir la résorption des lésions.

Conclusions: Chez les patients immunodéficients, l'infestation par *Entamoeba histolytica* est une des rares causes possibles de nodules pulmonaires non résolus par un traitement antibactérien adéquat. Nous recommandons de procéder à une biopsie pulmonaire percutanée et à une coloration spécifique des parasites lorsque cela est nécessaire.

Translated from English version into French by Suzanne Assenat, proofread by Céline Dubois, through

TRANSLATORS
WITHOUT BORDERS

Первичный амёбный абсцесс лёгкого у больного лёгочной аденокарциномой: клинический случай

Янь-Янь Лю, Юэ Ин, Чун Чэнь, Юэ-Кай Ху, Фэй-Фэй Ян, Лин-Юнь Шао, Сюнь-Цзя Чэн, Юй-Сянь Хуан

Аннотация

Справочная информация: Первичная лёгочная амёба встречается довольно редко, и в настоящей работе рассматривается случай заболевания 68-летнего мужчины после прохождения им курса химиотерапии против лёгочной аденокарциномы.

Описание случая: В октябре 2016 года в нашу больницу поступил мужчина в возрасте 68 лет с жалобой на кашель, который продолжался в течение 8 месяцев, а также на кровохаркание, длившееся в течение 1 месяца. Больному был поставлен диагноз «аденокарцинома лёгкого» и в 2012 году ему была сделана хирургическая операция без проведения химиотерапии. В марте

2016 года у пациента был выявлен рецидив рака, и больной подвергся курсу химиотерапии. После двух месяцев прохождения химиотерапии у больного появился продолжающийся кашель с выделением белой мокроты, а при проведении КТ грудной клетки у него был выявлен местный узел в лёгком. Врачи заподозрили у больного инфекционное заболевание лёгкого и провели эмпирическое антибактериальное лечение. Однако это не привело к облегчению симптомов, и в дальнейшем, при проведении чрескожной биопсии лёгкого были обнаружены трофозоиты *дизентерийной амёбы*. После приёма метронидазола у пациента появилось существенное облегчение симптомов и заживление областей поражения.

Заключение: В тех случаях, когда у пациентов с лёгочными узелками наблюдается иммунодефицитное состояние, а также при прохождении ими адекватного, но неэффективного антибактериального лечения, одной из редких причин инфицирования может являться *дизентерийная амёба* . При необходимости следует назначить чрескожную биопсию лёгкого с использованием специфического окрашивания для выявления паразитов.

Translated from English version into Russian by Liudmila Tomanek, proofread by Oksana Weiss, through

TRANSLATORS
WITHOUT BORDERS

Absceso amebiano pulmonar primario en un paciente con adenocarcinoma pulmonar: un estudio de caso

Yuan-Yuan Liu, Yue Ying, Chong Chen, Yue-Kai Hu, Fei-Fei Yang, Ling-Yun Shao, Xun-Jia Cheng, Yu-Xian Huang

Resumen

Información previa: La ameba pulmonar primaria es muy rara. Aquí se presenta un caso de un hombre de 68 años con ameba pulmonar primaria tras haberse sometido a quimioterapia para el adenocarcinoma pulmonar.

Presentación del caso: En octubre de 2016 un hombre de 68 años de edad fue internado en nuestro hospital por tos repetida durante 8 meses y hemoptisis durante un mes. Le fue diagnosticado un adenocarcinoma pulmonar y fue sometido a una intervención quirúrgica en 2012 sin recibir quimioterapia. En marzo de 2016 en el paciente se diagnosticó la recurrencia del cáncer y fue sometido a quimioterapia. Tras dos meses de tratamiento el paciente tenía tos constante con esputo blanco. Una TAC de tórax mostró un nódulo pulmonar primario. Los médicos pensaron que el paciente tenía alguna enfermedad pulmonar infecciosa y lo trataron con tratamiento antibacteriano empírico. Sin embargo, sus síntomas no se atenuaron y una biopsia percutánea pulmonar sucesiva mostró trofozoítos de *Entamoeba histolytica*. Tras la administración de metronidazol, los síntomas del paciente se atenuaron notablemente y las lesiones involucionaron.

Conclusiones: En los casos en que los pacientes con nódulos pulmonares se encontraban en un estado de inmunodeficiencia y fueron sometidos a tratamientos antibacterianos adecuados pero ineficaces, la infección de *Entamoeba histolytica* pudo ser una de las causas raras. Se recomienda la biopsia

pulmonar percutánea. Además, habría que administrar tratamientos específicos para eliminar los parásitos si fuera necesario.

Translated from English version into Spanish by Ribcamar, proofread by Mamagui4, through

TRANSLATORS
WITHOUT BORDERS
