# Supplementary figures and images for: Primary pulmonary amebic abscess in a patient with pulmonary adenocarcinoma: a case report
Source: Infect Dis Poverty. 2018 Apr 27;7:34. doi: 10.1186/s40249-018-0419-2 (PMC5921995; doi:10.1186/s40249-018-0419-2)

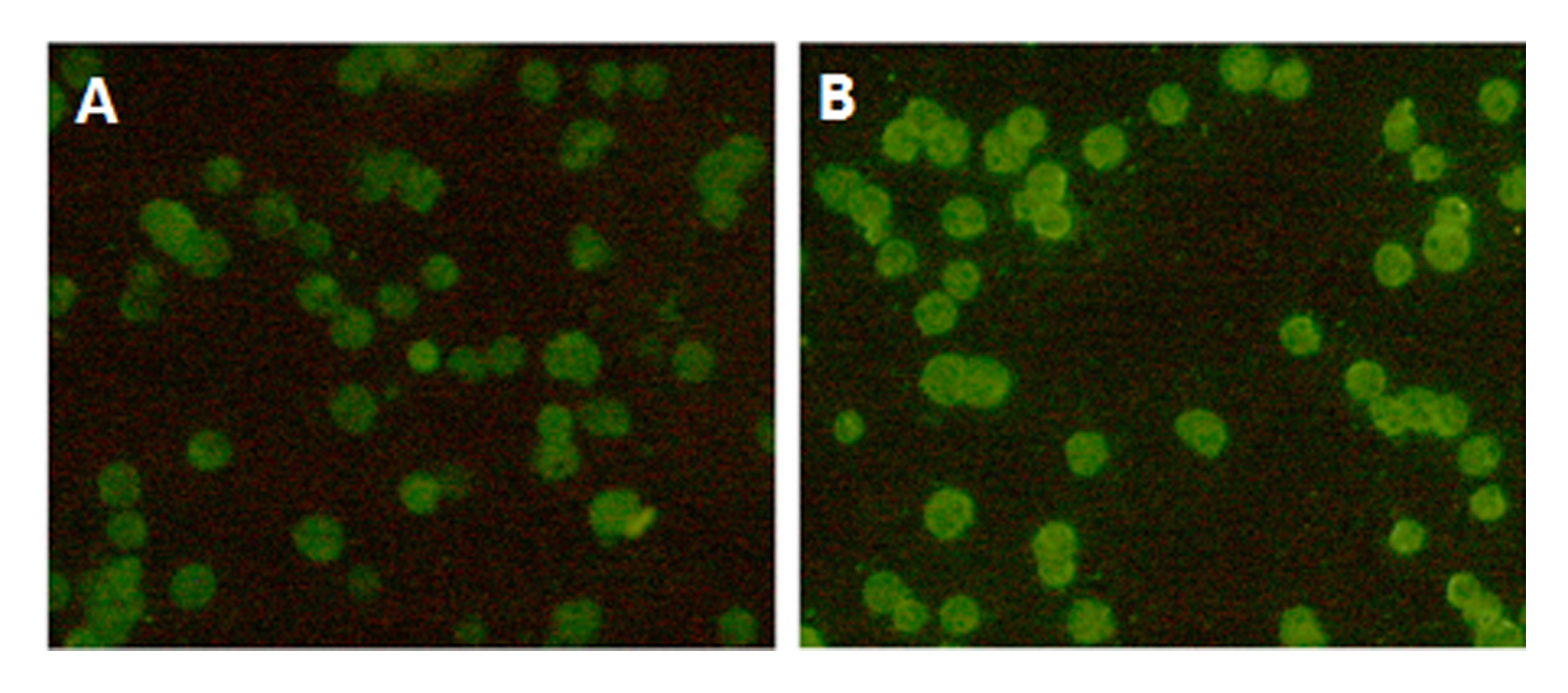

Supplement: Supplementary file 2 — Figure S1. The serum antibody of amoebic trophozoites was positive by Immunofluorescence assay. A, Negative control; B, Serum of patient. (TIFF 2286 kb) [file 40249_2018_419_MOESM2_ESM.tif]
